# Adaptive Water Management: On the Need for Using the Post-WWII Science in Water Governance

**DOI:** 10.1007/s11269-022-03373-0

**Published:** 2023-01-02

**Authors:** Peder Hjorth, Kaveh Madani

**Affiliations:** 1 Division of Water Resources Engineering, Lund University, Lund, Sweden; 2 Institute for Integrated Management of Material Fluxes and of Resources, United Nations University (UNUFLORES), Dresden, Germany; 3 The City University of New York Remote Sensing Earth System (CUNY- CREST) Institute, City College of New York, New York, NY USA

**Keywords:** Adaptive water management (AWM), Water governance, Transformation, Complexity, Uncertainty, Systems thinking, Integrated water resources management (IWRM), Sustainable development goals (SDGs)

## Abstract

Although the UN concluded, already in 1997, that water would be the most contentious issue of the 21st century, water governance is still confused, nearly everywhere. Even the severe impacts of escalating water bankruptcy and global warming have so far failed to incur a marked improvement in governance systems. The global community has adopted sustainable development as a common vision and guide for the future. Yet, the adoption of the underlying principles of sustainable development has been slow in the water sector and elsewhere. Despite the realization that water governance is a political issue, the near-universal neoliberal agenda tends to only employ technologic and economic solutions to address water problems. This paper presents a historical overview, from the end of the Second World War (WWII) and onwards, of events that could, or should, have had an impact on water management frameworks. It evidences some important consequences of the institutional rigidity exposed during that period. The paper also turns to the fields of science, policy, and management, to pinpoint failures in the translation of political rhetoric as well as new scientific findings into change at the operational level. It explores how an updated knowledge base could serve a quest for sustainable water governance strategies. It is argued that a persistent failure to learn is an important reason behind the dire state that we are now in. As a result, water management is still based on century-old, technocratic, and instrumental methodologies that fail to take advantage of important scientific advancements since WWII and remain unable to properly deal with real-world complexities and uncertainties. The paper concludes that when it is linked to a transformation of the institutional superstructure, adaptive water management (AWM), a framework rooted in systems thinking, emerges as a prominent way to embark on a needed, radical transformation of the water governance systems.

## Introduction

Around one-fourth of Earth’s population lives in countries that face an increasingly urgent risk of running out of water (Sengupta and Cai 2019). Many countries around the world are currently exposed to extremely high water stress (Shu et al. 2021; Berger et al. 2021) or are already in a state of “Water Bankruptcy” (Madani 2019; AghaKouchak et al. 2021) as a result of unsustainable use of freshwater resources and draining of non-renewable groundwater reserves (Rodell et al. 2009; Madani et al. 2016; Bozorg-Haddad et al. 2019; Escriva-Bou et al. 2020; Noori et al. 2021; Huggins et al. 2022). Major cities around the world, like São Paulo (Brazil), Chennai (India), Cape Town (South Africa), and Isfahan (Iran) have narrowly escaped the Day Zero during recent droughts (Sousa et al. 2018; Bischoff-Mattson et al. 2020; Ahmadi et al. 2020; Zaveri et al. 2021; Madani 2021a).

Globally, 78% of the jobs that constitute the global workforce are water-dependent (UNESCO 2016). The functioning of the world’s largest industry, agriculture, is extremely dependent on water to provide jobs to more than one billion people, who are mostly poor and living in poor countries. Agriculture is also the world’s largest water consuming sector and needs water to provide us with food. Energy is also highly dependent on water, making electricity production one of the major drivers of global water stress (Hadian and Madani 2013; Jin et al. 2019). It is, indeed, hard to imagine any human activity that does not depend on water (Savenije 2002).

Water shortage threatens biodiversity and ecosystems (Mirchi et al. 2014), which has major socio-economic and health implications for humans (Nabi et al. 2019). There are good reasons to be concerned. Massive flooding and wildfires have now made people, and thereby also governments, more willing to appreciate that what the nature is experiencing is not normal. Unless immediate and drastic action is taken, keeping global warming below a manageable level by humans would be impossible (Hjorth and Madani 2019; IPCC 2021). A forceful messenger has now come into play: the anthropogenic climate change is causing increasingly frequent, and progressively violent, water related problems in virtually any corner of the world (Levin et al. 2021; Madhukar et al. 2021; Roukounis and Tsihrintzis 2022; Bridhikitti et al. 2022; Montenegro et al. 2022). We are not only smashing record after record for warming and other impacts, but the world in which we live today has no recent parallel. Climate change is not a new problem in science. NASA, the US National Aeronautics and Space Administration, had established this threat as a reality, already in 1988 (Hansen et al. 1988). Still, this problem was, essentially, neglected until very recently. Nonetheless, the current policies—those already in force, not the many political promises of policies to be put in place at some future time—creare a credible risk of having to experience between 2 and 4 °C of warming (Pörtner et al. 2022).

Small-scale efforts will no longer be sufficient; what we need is a rapid, transformational change. Yet, governments seem to always have found other issues to be more urgent than water issues (Madani 2019, 2020), especially issues that might have an impact on the outcome of the next election. Somehow, they have not understood, or deliberately overlooked, the important role that water and the environment play in virtually every aspect of our lives.

Among the first things a water student gets introduced to in any elementary course, is the hydrological cycle – a system. Still, there seems to be very little of systems thinking in the structuring and functioning of water governance systems. It is now dawning to more and more people, that water is only a small part of a complex human-nature system-of-systems (Hjorth and Madani 2014; Madani 2019) with some “essential characteristics”, including uncertainty, bounded rationality, non-stationarity, limited predictability, indeterminate causality, and evolutionary change (Madani and Shafiee-Jood 2020; Madani 2021b). This open system-of-systems with multiple, overlapping hierarchies, is a kind of system that defies treatment by means of the old school analysis of current governance systems.

Even as the world gets increasingly complex, the natural laws are still valid. Numerical values are important. We use them to design and monitor our systems, forecast and project their future states, plan for the desired future, and develop mitigatation and adaptation actions. This is how we have built many water infrastructure and management systems that have enabled us to develop more, continue to grow, and improve global public health. But complex systems are highly dependent on the context in which they exist. It takes coordinates and quantities to properly characterize these contexts. So, using only numerical targets for the development of a complex system is a fundamental mistake. Likewise, indicator values should not be used for target setting. As Goodhart’s law tells us, “when a measure becomes a target, it ceases to be a good measure”.

We still suffer from the obsession with numbers and economic reasoning for explaining and solving socio-ecologic problems, while our quantitative methods are unable to catch the subtleness or beauty of real-world events. Thus, as the complexity of the world is increasing, we really have to rethink a great deal about the way that it’s governed. The problems are growing rapidly, and we urgently need to carefully reflect on what we will pass on to our next generation. Most likely, we have already created more than enough of problems for them to take care of.

Nature has now clearly shown us that it is not as stationary and consistent as we used to believe it to be. It cannot, as a whole, be reduced to a logic, that is in any way predictable. The feedback we have received from the nature in terms of unexpected variations of water quantity and quality as the result of anthropogenic activities, clearly stresses the need to modify our planning frameworks. We need to develop new water planning and management strategies that more properly account for uncertainties (Liu et al. 2019; Mirdashtvan et al. 2021; Lee et al. 2022; Sone et al. 2022), ranging from the uncertainties we are used to and wrongly think that we can quantify (e.g. flooding risk and return periods) to the uncertainties that we are not even aware of (i.e., the unknown unknowns and black swans). There is an urgent need to go beyond the predominant, century-old paradigms, which in turn, are rooted in the Enlightenment era (This was actually one of the main messages of the 1972 United Nations (UN) Conference on the Human Environment (Ward and Dubos 1972)!).

This paper calls for two major changes in water resources management. The first one is the required understanding that water management is an intrinsically political undertaking and that the prevailing political systems have an impact on the effectiveness of water governance. The second one is related to uncertainty and the required understanding that undecidability is a normal state in water management. The paper aims at diagnosing critical deficiencies of current practices and at showing some methods and foci that can support a struggle to find a way out of the current conundrum and get a grip on our growing water problems. To this end, the paper presents and discusses the post-WWII events that have (Table 1), or should have, had impacts on water governance. Among those are summons for betterment or change, from the UN, political events, scientific breakthroughs, and the tangible impacts of climate change. The discussion concludes that many of the increasing water management challenges around the world are largely the outcomes of decades of bad governance.

**Table 1:** Major post-WWII events with implications for water governance

**Year**	**Event(s) and implications**
1945	End of WWII; booming economy takes off; start of the “golden years”
1960s	Growing environmental and resource concerns; birth, environmental movement; general systems theory; living systems theory
1972	UN Conference on the Human Environment calls for new worldview; Limits to Growth: important application of system dynamics, poorly understood, but very important
1977	UN Conference on Water and lamentation of severe deficiencies in water resources management and in water and sanitation services in developing countries.
1980s	International Drinking Water and Sanitation Supply Decade; massive mobilization of resources produced lots of valuable experience; Prigogine finds living systems to be in “far from equilibrium conditions” and that order can emerge out of chaos, the importance of entropy.
1987	“Our Common Future” report: definition of “sustainable development” and guidelines for achieving it
1988	NASA establishes climate change as a fact and growing threat
1992	Dublin principles: an emerging roadblock; Agenda 21. The action plan for sustainable development
1996	Global Water Partnership (GWP) formed to launch a World Bank-style IWRM
1997	Rio + 5: water declared as the most contentious development issue for the 21st century
2000	The Millennium Declaration and the MDGs
2002	Rio + 10 finds the MDGs quite poor and launches alternative WEHAB agenda.
2003	UN-Water is launched
2012	Rio + 20 launches Sustainable Development declaration, finds a need to replace the MDGs with something better when end in 2015, prepares an inclusive interdisciplinary effort to develop new goals, the SDGs; UN finally walking its talk pays off, opens the door for alternative approaches.
2018	UN General Assembly declared an International Decade for Action: Water for Sustainable Development to accelerate the global efforts to address water challenges
2019	The COVID-19 pandemic forces governments to shell out financial support, in a magnitude not seen since WWII; It’s proven that transformations can happen quickly, if threats are severe enough

## Changes in the State of International Environmental Affairs: from Neoliberalism to Adaptive Management

### The Golden Post-WWII Era

In a way, the scene for world development post WWII was set in 1944, when in order to avoid new problems of the kind that the peace accord of WWI had caused, the victorious constellation of countries organized a meeting in Bretton Woods (Merrills 1977) to discuss what needed to be done to ensure a peaceful future. The end of the war marked an opening of the door to a new era. The misery and gloomy sentiment of the pre-war years were gone, and the sentiment was optimistic. There was a great willingness to build back better. The workforce was larger than before, as women had also been involved in the war industry. Science had made important advances during the war. The economy was booming, and living standards were rising rapidly. However, signs of negative impacts of the growing industry showed up in the 1960s. The existing development model was increasingly questioned, and the environmental movement grew strong. This forced governments to enact restrictions concerning pollution of air, water, and the environment.

In 1971, there was a more decisive sign that “the golden post WWII era” would be brought to a halt. In that year, the US expenses related to the Cold War had made the US surplus disappear, and President Nixon opted to abandon the Bretton Woods Agreement of keeping currency rates fixed relative to gold. Unsurprisingly, this wrought havoc to the currency markets, and created an economic downturn in Western countries. In 1973, the Organization of the Petroleum Exporting Countries (OPEC) issued an oil embargo targeting the countries that supported Israel in its war against an Arab alliance. Thereby, the cartel added to the downturn by causing significant increases of the oil price. Rodrik 1997, 1999) explains the reasons for the economic downturn and asserts that the immediate reason for it was the inability of governments to adjust their macroeconomic policies appropriately, in the wake of these external shocks. Still, this opened the door for a “false saviour”, the neoliberal agenda, which was eagerly adopted and pushed by Margaret Thatcher and Ronald Reagan (Caryl 2013). This agenda had serious impacts on the common sector, for example, on water resources management and governance, as one of its maxims was “more market and less state”. This caused a degradation of state capacity to govern, and a slashing of social expenses. Many government functions were privatized, and market thinking infiltrated deeply into public institutions.

The neoliberal principles were also enshrined in what became known as “The Washington Consensus”. It was an action agreed between the Washington institutions. Among those were the US government, the International Monetary Fund (IMF) and the World Bank, that all became heavy-handed pushers of the neoliberal agenda. This had dire consequences for many developing countries. It is argued that many developing nations are in debt and poverty partly due to structural adjustment policies enforced by international institutions that ended up increasing poverty rather than reducing it (Shah 2013). In Martin Khor’s opinion these policies not only resulted in trade liberalization, but also in deregulation of industry and privatization of state-owned services and industries, preventing governments from managing the basic services such as water, education, and health (Morrell 2005).

### Calling for a Systematic View to Enable a Brighter Future

By 1972, scientific advances called for a new worldview as they proved reality to be fraught with complexity, chaos, and uncertainty. However, these findings were so radical, that they were somewhat difficult for a layman to understand. Thus, the new findings had limited impacts. Nonetheless, the UN and the Club of Rome were both interested in the new worldview. The UN launched a “Conference on the Human Environment in 1972 (Ward and Dubos 1972), and the same year, the Club of Rome presented its report on development and our resource base “Limits to Growth” (Meadows et al. 1972). Thus, the first global warning about the course of the development was issued at this UN conference. There, the new worldview was well represented, and it was found that the world would be in deep trouble by 2000, if the rapidly increasing resource use trends were allowed to continue unabated. Several new ideas were discussed and/or applied. The notion of sustainable development was mentioned, and the conference showed concern about the development of climate change and biodiversity loss. The discussions were summarized in an unofficial report “Only One Earth” (Ward and Dubos 1972). It reflected the idea that 400 years of “The Age of Enlightenment. Science” was enough, and that its whole framework of thinking needed to change, and that we needed a Copernican Revolution-type event that could bring a serious paradigm shift. It was argued that our thinking had been based on the measurements and analyses of discrete particulars, but then there was a new realization, that there was also a need to focus also on smaller things, different processes, and webs of interrelationships. These things had been found to be equally real and scientific as the measurable, the vast, and the powerful.

The Club of Rome report had been initiated by a concern that our profligate resource use might create serious resource problems down the road. As the study was based on systems thinking, many people didn’t really understand its message, which essentially confirmed what the UN conference had found. However, Limits to Growth went further, and analysed what would happen if we should be able to pass the first resource obstacle. The report stated that the next barrier would be the limited capacity of the environment to assimilate all the waste that was created by our civilization (climate change is a manifestation of the workings of the second barrier). If we managed to clear that barrier, we would run into a third, and so on.

Overall, these two events called for a new worldview, a systemic one, with intrinsic complexity and uncertainty, based on an understanding of the human civilization as an integrated part of the global ecosystem. That message was difficult for most people to stomach. In a way, these results corroborated what Marcel Proust and Albert Einstein had previously suggested. Proust tried to tell us that “The journey towards a regenerative culture is about embracing all of Nature as the ground of our being - seeing ourselves and thereby everything with new eyes” (Wahl 2017). Albert Einstein also reminded us that “A human being is a part of the whole, called by us ‘Universe,’ a part limited in time and space. He experiences himself, his thoughts and feeling as something separated from the rest—a kind of optical delusion of his consciousness.” When we begin to “free ourselves from this prison,” as Einstein phrased it, then we expand our consciousness to “embrace all living creatures and the whole of nature” (Haymond 2018). Yet, Einstein’s wisdom has remained hard to understand for the many.

### The Poor State of Water Affairs

The next warnings about development problems were issued by the International UN Conference on Water in 1977. Here, the world was warned that water resources management was embarrassingly inadequate in most countries. There was also a serious reminder that water resources assessments, in most places, were so haphazard, that they could not be used in a meaningful way, as a basis for water resources management. The conference also stressed the urgent need to establish properly integrated and coordinated approaches to water management.

In addition, the conference noted the appallingly poor state of water supply and sanitation services in most countries of the developing world. Thus, the conference issued an urgent plea to the international community to join hands and rectify this serious problem until 1990. This plea had a dramatic impact. The UN declared the 1980s to be the International Drinking Water Supply and Sanitation Decade (IDWSSD). This really mobilized the international donor community, that went all in to solve the “water, sanitation and hygiene (WaSH) problems within the allocated time frame. Several countries did also make significant efforts to extend or rehabilitate the water and sanitation services in their respective countries. Thus, there was nothing wrong with the energy that went into the efforts made. However, there was a problem with the know-how. Consequently, the Decade missed its target by a relatively wide margin. Unfortunately, the good will demonstrated during the Decade, rapidly petered away, once it ended. Nevertheless, the Decade provided ample opportunities to make new experiences, good and bad. After the Decade, those experiences were distilled and presented in “The New Delhi Statement” (UNDP 1990). Thereby, the sector was provided with an excellent guide to water and sanitation provision in developing countries. But many development aid organizations seem to have turned a deaf ear to its messages, Therefore, we still see many failing water and sanitation projects. As we shall see, the tide turned in 2012, but there are still many boats, that have failed to adjust their course to this change of tide and are blindly steering into the wild.

### A New Human Agenda: Sustainable Development

Inspired by the 1972 UN conference, the UN established a World Commission on Environment and Development. It was charged to develop a roadmap to sustainable development in the 21st century. The Commission launched its report “Our Common Future” in 1987 (WCED 1987). It caused very heated debates, as it argued for a paradigm shift. The messages from 1972 had taken effect: the severe problems that could emerge, if we followed the current development trajectory, would be too harsh. Thus, the report called for significantly more democratic and egalitarian states. Here, sustainable development was described as “development that meets the needs of the present without compromising the ability of future generations to meet their own needs.” In this description sustainable development is a paradigm in which environmental, societal, and economic considerations are balanced in a harmonic way, in the pursuit of improved quality of life. For example, sustainable development strives to provide healthy environments able to provide food and resources, including safe drinking water and clean air for all citizens. Sustainable development was also presented as a development that produces equality within and between generations. If we consider politics, “in its broadest sense”, as “the activity through which people make, preserve and amend the general rules under which they live” (Heywood 2013), we can see that sustainable development, in fact, is a political quest for the rebalancing of society into a more considerate, egalitarian, and democratic state. So, not surprisingly, not everyone agreed with the report, as many people wanted to stay in their current comfort zones.

In June 1992, the UN organized a World Conference on Environment and Development in Rio de Janeiro, Brazil. The major task was to establish “Agenda 21”, a roadmap for sustainable development in the 21st Century. The Conference managed, in fact, to agree on such an agenda. It consisted of 21 chapters, where Chap. 18 was devoted entirely to water. In addition, the Conference established two conventions, one on climate (CCC) and one on biodiversity (CBD), each with recurring Conferences of the Parties (COPs), to promote and monitor governments’ actions to address them.

To many people, there were two major problems with sustainable development. The first was that it requested us to have a feeling of solidarity, not only with our neighbours and fellow countrymen, but also with nature. That was something unheard of. The second problem affected mainly the scientific community. The concept went beyond their beloved scientific methods, as it involved ethics and an undefined timeframe. Thus, neither calculations nor mainstream “rational reasoning” would be able to prescribe or predict the best trajectory for a transformation into sustainable development. Another controversial point was that it declared that sustainable development was strongly tied to a harmonious interaction between the social, environmental, and economic aspects, which pushed the economists off the pedestal, that they had been enthroned on. In addition, sustainable development was presented as a context-dependant process rather than some fixed goal (Hjorth and Bagheri 2006; Hjorth and Madani 2014). Within the water sector, the notion of sustainable development as an oxymoron was widely adopted. Thus, it was seen by many as a self-contradicting concept that could safely be ignored.

### A Counter-revolution?

A few months before the above Conference, in January 1992, Ireland organized an International Conference on Water and the Environment. Here, the delegates were experts nominated by their countries or by international organisations. The conference developed a statement “The Dublin Principles”, which contained four principles for integrated water resources management (IWRM), one being that water should be treated as an “economic good”, i.e. as a commodity to be bought or sold in a market (WMO 1992).

Consequently, the World Bank created two satellite organizations, namely the Global Water Partnership (GWP) and the World Water Council (WWC). In 1996, the GWP was formed to launch a World Bank-style IWRM formula (GWP 2000), with the Dublin Principles as its core principles. WWC was charged to monitor and support the spread of the IWRM concept. The monitoring manifested itself in recurring World Water Forums, where delegates would present and discuss the uptake of the IWRM idea. As noted by Biswas (Biswas 2004) and many others, the heavy backing of the concept by international and regional development banks made it spread like a wildfire, to become almost the lingua franca within the water development community. As it turned out to be an unworkable proposition (Madani and Shafiee-Jood 2020), seemingly entangled in technical and institutional intricacies, rather than solving practical problems (WWC 2018), it essentially served, for a long time, as a wet blanket thrown out over alternative approaches to water governance.

At the 5-year sequel of the UN conference (i.e., the 1997 UN Special Session of the General Assembly to Review and Appraise the Implementation of Agenda 21 in New York), the cooperative spirit demonstrated in 1992 had vanished. Then, countries had embarked enthusiastically on Agenda 21 activities. However, by 1997, these activities had lost much of their momentum. Thus, the only issue the delegates managed to agree on was, that water would be the most contentious issue in the 21st century. The basis for this decision was i.a.a sector overview produced jointly by WMO and UNESCO (Kjellén and Mcgranahan 1997).

### A Reminder: Millennium Declaration

By 2000, the UN found a need to issue a reminder concerning sustainable development. A Millennium Declaration was developed to this end. It essentially reiterated the key messages from Agenda 21. It was also decided to let the Declaration be accompanied by a set of Millennium Development Goals (MDGs), to be achieved by 2015. The elaboration of the goals was entrusted to an expert group, which produced eight goals and a score of targets. None of these goals or targets mentioned water. As could be expected from experts, the goals and targets were all numerical, and they came without any associated priorities.

At the 10-year sequel of the UN conference (UN 2002), it was concluded that the MDGs were poorly conceived. Thus, the delegates produced an alternative – the WEHAB Agenda, which came with priorities and action plans for the priority areas. In this acronym, W stands for water and sanitation, which got the highest priority. On places 2–5, WEHAB included, in order of priority, environment, health, agriculture, and biodiversity. However, most countries opted to stick to the more familiar MDGs. Still, the WEHAB agenda made the UN realize that it needed to add some water goals to the MDGs. In 2003, the UN established UN-Water as an interagency mechanism to coordinate the efforts of United Nation entities and some other international organizations working on water and sanitation issues. UN-Water then became the coordinator of the ‘Water for Life’ International Decade for Action (2005–2015), to help the world meet the 2000–2015 Millennium Development Goals’ sanitation target.

### IWRM: A Search in Vain

OECD (OECD 2011) reports a study of water governance in 17 OECD countries, 45% of its members, and found the water governance system to be deficient in all of them. Although there is a diversity of contexts, it was found that a number of common challenges exist. Those include fragmented institutional structures, limited capacity, particularly at the local level, unclear roles and responsibilities, and questionable resource allocations. These problems were often found to be rooted in misaligned objectives and poor management of interactions between stakeholders. Lack of long-term strategic planning was, together with poorly drafted legislation, also to be blamed. These governance problems were, unsurprisingly, found to be a cause of an ongoing degradation of the natural resources base. The study noted a recursive and self-perpetuating relationship between traditional institutions, which impacted their discourses and practices and thereby hindered transformative change. Those social and cultural constraints were found to be a persistent problem. Thus, it seemed that the traditional institutions confined their strategy discussions to the common cocoon, and what was made public was like bikinis, in that “what they revealed was suggestive, but they concealed the vital”, to use a metaphor from Levenstein (Mahajan 2007).

Thus, it’s no wonder that a belief, that the right institutions will lead to the goal of good water governance, has made many actors make an endless search for the ‘institutional Holy Grail’. This trend has frequently shown up in the application of the IWRM concept, where this has been equated with good water governance (Allan and Rieu-Clarke 2010; Lautze et al. 2011). Due to the heavy backing by the World Bank and its accomplices, IWRM has been recognised around the world as the solution for solving a country’s water problems, although as Allouche (2016) remarked, the perception of IWRM as a universal model for water governance had been increasingly questioned. In this case, one really needs to, as the 2017 World Development Report (World Bank 2017) suggested, investigate why some bad policies can endure.

### From the MDGs to the SDGs

Unsurprisingly, it had become evident already in 2012, that the MDGs would miss its targets by a wide margin. Thus, at the Rio + 20 event, which took place that year in Rio de Janeiro, the birthplace of Agenda 21, it was decided that there was a need for something to promote sustainable development post-2015. However, this time, it was understood that the UN needed to “walk its talk”. Therefore, the development of a new declaration and an associated agenda was to be made in a much more inclusive way, and in a thinking mode that corresponded to the spirit of Agenda 21. To operationalize this idea, there were multi-sectoral, inclusive working groups established to produce a post 2015 Sustainable Development Declaration and accompanying Sustainable Development Goals (SDGs). This time, it was also realized, that the progress on the goals needed to be monitored in a more rigorous and meaningful way, than had been the case with the MDGs, that had essentially been monitored by means of self-reporting. The documents produced were published under the highly inspiring banner of “The Future We Want” (UN 2012). The Sustainable Development Declaration and the SDGs were launched in 2015 (UN 2015), adding to the momentum that the 2012 event had created. Noting the essential role of water, the UN General Assembly declared 2018–2028 as the International Decade for Action “Water for Sustainable Development” to accelarte the efforts to address global water challenges.

These events had a remarkable impact on the development community, and alternative approaches, that had previously been suppressed by a dominant mainstream, were allowed to surface and show their merits. Many actors had, eventually, understood the spirit of Agenda 21. Relative to the post WWII science discussed, adaptive management ticks all the boxes. It is solidly based in systems thinking, it respects the inherent uncertainties and, primarily attempts to find solutions that are “good enough”. It makes use of extended peer groups, with inclusive involvement and collaboration of local stakeholders, which effectively ensures that that the project context is properly accounted for. In line with Nowotny et al.’s suggestions (2001), it does not rely on any scientifically determined values to assess the validity of the results, but relies on the common sense of the joint partners. Any solution is seen as provisionary, as there is an appreciation of the constantly changing global environment.

It also fits well with the OECD report, which points out the local limb as a major obstacle to adequate water governance and, in particular, the poor management of interactions between stakeholders. As the collaborate methods support learning and empowerment of the actors involved, it can also respond to the need expressed by a recent GAR report (UNDRR 2021), that the UN Office for Disaster Risk Reduction recently published. It aims to raise awareness of the risks posed by periodic deep droughts. However, rather than focusing on particular methodologies or cases, it argues for a systems-based management, that can serve to build a capacity of systems and people to help them imagine, adapt and co-produce a sustainable and equitable future. The report emphasizes a need to substantially increase the likelihood that particular, local characteristics get adequate attention. It does not aim at a single target but aims at creating a distributed and agile network. This is seen as an amazingly relevant response to the challenge, that we now must really be focusing on. It is asserted that this might be one of the most important issues to cater to if we want to ensure a reasonably liveable future. It is important to help everybody understand what can be done to prevent dangerous incidences as far as possible, and also to equip them with knowledge about adapting to and sustaining the incidences that we fail to prevent.

## The Science Behind the Scenes

### Systems Science Impact on the Club of the Rome Report

Jay Wright Forrester, a systems scientist at MIT and the founder of System Dynamics, was among the first to apply systems thinking to real world problems (Forrester 1971). The Club of Rome report was undertaken by some of his students, and was based on the world model, that he had developed. This was a very simple model that contained only five state variables: (1) total human population; (2) total persistent pollution; (3) remaining non-recoverable natural resources; (4) total capital investment; and (5) fraction of capital investment allocated to the agricultural sector. Forrester wanted to demonstrate, in this simple way, that physical systems are inherently constrained. In our world, production of goods (especially food) on this globe has limits, determined by the available resources. He could also show that energy constraints will prevent production from growing indefinitely. The work was built on the general systems thinking developed by von Bertalanffy (1950, 1968). Thus, it attempted to show what would happen to the world as a consequence of the interactions between the five variable elements. Based on an assumption that the then trends would remain the same, it was possible to get a coarse estimate of what would happen to the variable components. In the current case, it became quite clear that there would be some serious resource problems by the end of the century.

The report acknowledged that, for a while, the growth of various parameters such as world population, resource consumption, and environmental pollution may appear to defy physical limits. But soon, the systemic feedbacks kick in, and a marked decline or even collapse of the industrial society is the only option for the world system to return to more normal conditions. Thus, there is a delay between the temporary overshoot and the ultimate collapse, due to the various time lags in the interplay between causes and effects in resource depletion. Assuming a different trajectory, that evaded the resource problems, made the model indicate a new problem – the problem to handle all the waste produced along that trajectory. If it were possible to evade that problem, there would be a problem to feed the global population. There are limits to how much we can grow. The “green revolution” has been hailed for the productivity increases it was able to generate, but this came at a cost of the use of two of our most critical resources – water and energy. Plant growth is a natural process, and cannot be rationalized, as electronics have been. In addition, much fertile soil has been ruined by salinization and waterlogging respectively. New land has mainly been developed by deforestation, which has serious impacts on climate and biodiversity.

Th developed model suggested that it is possible for our civilization to “overshoot” and go beyond planetary limits for a limited period of time. However, no society, or even the human race as a whole, can live beyond their means for a prolonged time. No matter what we do, the improvement of our economic welfare, and the excessive encroachment on global carrying capacity will only be temporary. But Limits to Growth was poorly understood. It was perceived as a forecast, as this was what people were used to. The report was either ignored or ridiculed. It also sparked polarizing debates among academics (Gardner 2004). The book suggested that something as big as a planet, which in our human eyes, seems to be fathomless, can be compromised. That was a profound contribution.However, economists, business leaders, and politicians didn’t see the report that way (Meadows 1988). They only saw scaremongering forecasts. It was banned by the Soviet Union and in the US, The Nixon White House denounced it (Meadows 1988). The possibility of global collapse was so terrifying to many people that they badly wanted to find errors in the analysis. Others just rejected this possibility. “You wouldn’t think such simple conclusions would stir up much fuss, but the fuss was incredible. The storm went on for years,” Meadows (2001), one of the co-authors later commented.

The researchers published a follow-up report after twenty years. By then, they had refined the model, and it still supported the old conclusions. In this report, it was also discussed what people had misunderstood in the first, and the authors tried to clarify the issues. However, with limited success. There was a second follow-up in 2004. Again, the authors were able to show that the model was valid. The main conclusion was that the global community had wasted valuable time, and that it now would take much more determined action to avoid the problems that were lurking ahead. Even this finding was mostly denounced or neglected.

### Injecting Science into Management

Following the 1972 UN conference and the publication of “Limits to Growth” (Meadows et al. 1972), the ecologists got interested in adaptive planning. They already had some understanding of systems, as the concept of ecosystems had been in use for a couple of decades. At the outset, Holling (1978), Walters (1986), and a few others, recognized the importance of systems biology and ecology in determining goals for environmental management. They realized that these systems were far too complex to be amenable to analytical methods. Thus, they opted for tinkering, the age-old methods to improve on things, or, as it is currently known – trial and error. Adaptive management, was, thus, adopted as a comprehensive scientific approach to environmental management serving to guide a trial-and-error process. It was suggested that this process could be captured in three principles: (1) It is experimental, and disagreements should, whenever possible, be articulated as hypotheses that could either get confirmation or get falsified; (2) It models natural systems as being multi-scalar and hierarchically ordered. Thus, ecological systems can be seen as nested systems, where larger systems change much more slowly than their nested subsystems; and (3) It is tied to a specific place, in the sense that all observations and all measurements—as well as policy formation—are first based on the specifics of a particular site. Larger systems are also viewed and understood from an inside-out perspective.

It soon became clear to them that human beings had important impacts on their ecosystems. Thus, they incorporated humans in their planning (Holling 1973, 1978; Walters 1986). Later, ecologists started to use the term “Socio-Ecological-Systems”, SES. The SES applications are based on an insight that the ability to predict future key drivers that influence an ecosystem, as well as its behaviour and responses, is inherently limited. Thus, management must be adaptive and have an ability to change management practices, as new experience and insights suggest a need for correction. Adaptive management is thus a systematic process to continually improve management policies and practices, by means of the learning provided by the outcomes of implemented management strategies.

That approach, thus, represented an effort to inject science into management. As the impacts of the human enterprise became increasingly obvious, some actors found a need for a new approach to resource management. Levin (1998) understood the increased complexity when humans are interfering with ecological systems, and he became one among the first to explore complex adaptive systems. Others started to experiment with adaptive collaborative management (Buck et al. 2001), which includes both the principles of adaptive management and embedded science, and the creation of public process to achieve deliberation and social learning, that could help boost attempts to protect and restore ecological systems. As they worked on these approaches, the adaptive managers started to understand that research into systems of nature, understood holistically, would inevitably lead to reconsideration of goals, values, and priorities. This insight implied a move away from the positivist scientific approach that had been favoured by the mainstream scientists. Lee (1993) accentuated this shift, as he introduced the process-based approach of the American pragmatists’, particularly Dewey (1927) idea of social learning through deliberative discourse. Here, adaptive science learns from experience, and ensuing public discourse can create changes in values and priorities.

### Finding Traditional Science Insufficient

“Our Common Future” introduced some new concepts into the development discourse in 1987, and the World Conference on Environment and Development repeated and emphasized them in 1992. For instance, evolution, equality, and harmonious development. Such concepts defy scientific knowledge, and transgress the domain of traditional science, with its roots in the Enlightenment. In that kind of “normal” science, uncertainty is abstracted away, and values don’t enter at all. Foundational issues about the relevance of an application of a particular method are never raised.

It is fair to claim that the emphasis on sustainable development was a reaction to the fact that scientific expertise had led the world into policy dilemmas for which it had no solution. Now, the need to adopt a new worldview was strongly emphasized, as was the need to tackle deep uncertainty (Kasprzyk et al. 2012; Walker et al. 2013; Marchau et al. 2019; Brown et al. 2020), and sometimes ignorance, as well as the ethical conundrums intrinsic to core policy issues.

The concept of “Sustainable Development” could have been less enigmatic to the many, if they had been familiar with Dewey’s (1927) theory of democracy, which does not require achieving at one stroke the greatest welfare for all (and he emphasized that there can’t be a formula for determining that), but it only demands our best efforts to incrementally move towards that ideal goal, through social change—and he had found that there is no lack of evidence that democratic societies have been able to accomplish such transformations.

As Lyotard (1984) had explained, we had entered a new era, postmodernism, where traditional science was unable to serve as our only guide. In 1993, Funtowicz and Ravetz (1993) suggested that a science appropriate to this condition must acknowledge that real world problems are affected by inherent uncertainty, incomplete control, and the existence of multiple legitimate perspectives. Thus, they claimed that policy issues, even in the early stages, could be better handled, if they draw on experience beyond that of the traditional participants, i.e. experts, science advisors, and politicians. An extended peer community would be needed to ensure an insightful dealing with these issues. They characterized traditional science as Mode 1 science, and the new approach as Mode 2 science. Thus, the traditional analytical worldview, represented by Mode 1 science was complemented with an approach that is synthetic, holistic, and humanistic. Systems involving human agency were seen by them as emergent and impacted by reflection and contradictions.

In 2001, Nowotny et al. (2001) added to the ideas of Funtowicz and Ravetz. They claimed that science had proven itself to be more efficient than common sense in producing tangible results. In their view, humanity was facing radical uncertainty and even ignorance, as well as inability to deal with ethical issues that lied at the heart of scientific policy issues. Thus., they concluded that quality assurance of the scientific information provided for policy decisions would be impossible to establish without new ways to provide such assurance. Consequently, they suggested that we use social robustness as a measure, for which they defined five crucial properties: (1) The notion is relational, rather than relativistic, since it can only be assessed with regard to some particular context; (2) Social robustness and stability can only be achieved after enduring processes and iterations; (3) We must make a distinction between social robustness and the acceptability of knowledge claims. Still, however, they remain inseparable; (4) Robustness (and why not sustainability?) of research on complex issues can be reached only if science is open to, and improved by, social knowledge; and (5) To become socially robust, knowledge needs to be empirically grounded and duly verified, by means of frequent tests and improvements. They also added that, as issues influenced by humans tend to be emergent, we must also realize that the related research will never be strictly finalized, but stays rather open-ended.

Gigerenzer (2015) was inspired by the new thinking documented at the 2012 Rio + 20 event. He then believed above all in the power of simple rules in the real, unfathomably complex world. “Probability theory is the best thing in a world where you can measure the risks exactly and the parameters are not too complicated. But for most problems it just provides another illusion of certainty, and becomes part of the problem,” he argued. Until now, he continued, the rationality of reductionist natural-scientific research has been taken as a model for the rationality of applied science and intellectual and social activity in general. He was convinced that science can no longer evade issues such as the management of irreducible uncertainties in knowledge and in ethics, and the recognition of different legitimate perspectives and ways of knowing, respectively. Thus, he concluded; we need to develop a new practice, more akin to the ideals of a democratic society, characterized by processes of extensive participation and toleration of diversity. As sustainable development compels us to also recognize our obligations to future generations, to other species and indeed to the global environment, he claimed that science also needs to expand its scope.

### Other Major Scientific Contributions that Facilitated the Paradigm Shift

von Bertalanffy (1950, 1968) played a major role in the exploration of system properties and managed to generalize his findings and launched a theory of general systems, which included humans as well as other living systems. This work made us aware of the intricate, complex web of impulses that goes between the components of a system and in the end, determine the behaviour of the system. Prigogine (1981; 1997) and Prigogine and Stengers (1984) worked at smaller scales. Their research, that had started already in the 1950s, resulted in increased understanding of complexity, chaos, and uncertainty. Prigogine proved that all living systems are in far-from-equilibrium conditions, which is contrary to the old belief that equilibrium was a desired and harmonious condition. A system that can maintain itself in a far-from-equilibrium condition was classified as a dissipative system. Such systems need to struggle to remain in far-from-equilibrium. Thus, a living system needs an input of energy or matter from its environment, both to develop and to maintain itself. This work earned Prigogine a Nobel Prize in Chemistry. In 1997, he published a book (Prigogine 1997) in which he summarized and explained his results concerning the inherent uncertainties in natural processes, and he gave the book the telling title “The End of Certainty”.

Simon (1955, 1962) was a pioneer in several modern-day scientific domains. Among these were artificial intelligence, information processing, decision-making, organization theory, adaptive management, and complex systems. In Simon (Simon 1962), he was among the earliest to map the architecture of complexity, and he explained how the interactions between system components compose an intricate web, which forces the systems out of the realm of what could be studied by means of analytical methods. This, in turn, implied that such systems were characterized by an inherent uncertainty. He also pointed out some weaknesses of real-world humans and remarked that these humans had little in common with the stereotype of human used in economic theory. Thus, Simon may well have been the first behavioural economist in modern times. His work is also highly relevant to adaptive management.

He identified two human characteristics, that are critical to human decision-making, namely bounded rationality and a limited span of attention (scope or frame). This effectively killed the “economic human”, the stereotype of human used in virtually all economic reasoning. Concerning planning, it means that people don’t optimize, instead, they search for a good enough solution. The limited span of attention means that people don’t know, and don’t observe everything, as the “economic human” does. Importantly, the span of attention often differs between individuals, making them have different worldviews. These are problems that need serious attention in efforts to make people agree on a common agenda for action, such as in adaptive management of a watershed. Together with James March (March and Simon 1958), he provided an empirically-based understanding of human behaviour and coordination, and set up core scientific criteria for management and organization of research. This work came with an explicitly stated goal of ‘replacing fancy with fact’. Thus, the book called for planning frameworks, that aimed at good enough solutions, and had a focus on what should be included, and what could reasonably be left out of consideration. Within planning, this task is known as scoping, where stakeholders have to agree on the objectives of the planning, and then make a heuristic list of factors that are of importance relative to the established objectives. Concerning individuals, it is mostly known as “framing”. From the above, it is also clear that planning should be undertaken with the widest possible representation of all legitimate interests. Problems that may create difficulties are, for instance, that the knowledge base is incomplete, characterized by uncertainties, and often contested; that the perceptions about the nature of the problem are vague; that the view on potential solutions diverge, at least in the early phase; and sometimes, that the institutional setting does not have well-defined procedures.

### Promoting Adaptive Management in the Water Domain

Claudia Pahl-Wostl was an early proponent of adaptive management in the water sector. She has been a persistent advocate for adaptive management, but for long, she represented a rather lone voice in the desert (Pahl-Wostl 1995, 2007a, b, 2015, 2017). In relation to the clearing of ice in 2015, she published a book (Pahl-Wostl 2015), in which she attempted to develop a theory for how water governance can transform from what she calls “technocratic approaches and instrumental management” to an approach to “foreground the ‘human dimension’”. She also tried to develop a theory that describes under what conditions, and in what way, governance can transform and adapt to a more flexible and participatory planning framework, which she labels as MTF, short for ‘Management and Transition Framework’. Here, she bridged the gap that has existed between AWM, which essentially concerns locally based planning, and the institutional superstructure, within which the learning on the ground needs to “trickle up” in order to impact the entire water governance system.

She has found that it is informal social learning that produces new ideas and emphasizes agency. The formal policy processes are where these ideas are then codified into a new regime. Both processes are important, and a tight connection between them is crucial for responsive change. Given the importance of learning and path dependence, her book presented a social learning model that included single, double and triple loop learning, where the latter is seen as being particularly critical for governance transformation. In Pahl-Wostl (Pahl-Wostl 2017) she pointed out that water related problems often can be attributed to governance failure at multiple levels of governance, rather than to problems with the resource base itself. Thus, to improve on water governance, we need to put strong, emphasis on processes of transformation and change, from the ground up, which she saw as a key aspect of moving towards more sustainable water governance and management. She also asserted that, the challenge is to develop an understanding of the processes of change in policy implementation, and of social and societal learning, rather than in creating blueprints for system architectures, which appears to be the mainstream approach, that often ends up in nothing more than simplistic panaceas for governance reform.

## What We Learned and the Path Forward

### Context Matters

We now understand that there are no universal formulas, that can be applied everywhere. A successful project needs to cater for the needs of the local environment, and to the local people, that are supposed to operate or benefit from it. It has to fit with both the terms of humans and those of nature. Such projects cannot be designed in distant offices, or with the help of “suitcase consultants”.

Lee (1999) was a kind of generalist, but he was among the first to consider the role of adaptive management in the water sector. He thought that it would make more sense to think of system management as managing the people who interact with the system. He realized that this focus raises questions to which there are few reliable answers, but he thought that they can be explored, by means of experimentation. He also made the interesting observation that “many public policies are grounded in anecdotal knowledge, especially those enacted by legislatures, referenda, and general-purpose governments. From this perspective trial and error is an unusually systematic way to learn.” As he was well experienced in systems thinking, he noted that the complexity of a waterscape could actually mean that even simple steps may yield surprising outcomes - and science can be an efficient help in recognizing and diagnosing surprise. In principle, this makes us able to learn, over time, how management does and does not affect outcomes. He understood water governance as related to the goal of sustainable development and realized that reliable knowledge of natural systems used by humans is essential, if a sustainable society is to be achieved.

Management of water in a larger area poses, what Churchman (Churchman 1967) described as, wicked problems. These are often difficult to pinpoint, and they defy analytical approaches. In addition, they can never be solved once and for all. As Jentoft and Chuenpagdee (2009) noted in their work on fisheries and coastal areas management, a “good enough” solution can best be found by means of the judgement of stakeholders within a process that is experimental, interactive, and deliberative. Here, it should be clear to the participants that there are limits to how rational and effective a management (or governance) can be. Thus, there needs to be a lively, interactive communication among the stakeholders within the different stages of the process (Herman et al. 2014; Hadjimichael et al. 2020). Ideally, the process should be inclusive and self-correcting, to make it alive and changing as learning goes on. These processes essentially follow what Herbert Simon (Simon 1957) suggested in his book “Models of Man: Social and Rational”, where he stated that, most real world situations, if not all, are poorly structured, and for each of them, we need to decide how to best “frame” it, where the frame serves to delineate the part(s) of the problem where we should focus our attention. Thus, there is a need to search for a frame that is accepted by all participants, in order to find an acceptable solution to a problem at hand. He also stressed the importance of the balance between long-term and short-term aspects and held that it would be very difficult to delineate a workable governance strategy if this aspect is neglected.

### From Water Management to Inclusive Water Governance

Within the water sector, it is, in particular, the AWM that has gained currency. In addition, it has become increasingly recognized that water resources management is a political issue. Thus, there is now a tendency to speak about the water management task as water governance. There is also a dawning understanding that AWM, which starts from the ground, makes much more sense than the dominant IWRM approach, which has become mostly a prescriptive, top-down methodology, that never managed to make ground contact. Thus, we have a burgeoning flora of AWM varieties. These seem to differ mostly in how power is shared among the different stakeholders within a particular application. There are grounded reasons to assume that the “best” outcomes are achieved when the project owners are allowed to have the final say. However, most funding agencies tend to have some more or less hidden agendas, which make them unwilling, or even unable, to give up their reins on the planning processes. Thus, the AWM varieties have mainly been developed to cater to the needs that various donors or funding agencies have to maintain a certain control of the planning process and its outcomes.

The World Bank, for example, has dipped a toe into adaptive management, starting in 2012, when it allowed some of its staff to start experimenting with what was first called “Doing Development Differently” or DDD (DDD Workshop 2014). This was done as part of a program “Building State Capacity”, run by Harvard University. Its rationale is said to be that too many development initiatives fail. This had made the partners realize, that genuine development progress is complex. Problem solutions are not simple or obvious, and those meant to benefit most lack power, those, able to make a difference, are disengaged, and overlooked political barriers turn up too often. Most development initiatives were found to fail to address such complexity and embark on irrelevant interventions that have little impact. This work is reflected in the World Development Report 2017 (World Bank 2017), which deals with issues like: Why do carefully designed, sensible policies often neither get adopted nor implemented? In case they are, why is it that they often fail to generate the intended development outcomes. And why do some bad policies endure? As one of the important findings, the report states that institutions should be judged, not only by their looks, but also by how they function. It also admits that some of the Bank’s core beliefs have been faltering on several occasions. This concerns i.a, the dictum of international tendering for project inputs. Local sourcing strengthens the local infrastructure, which can promote the sustainability of a project. Another example is the idea of “Best Practices”. As mentioned, context matters, and a blueprint from a distant, different environment is likely to be a poor fit.

The theory of social change that John Dewey (Dewey 1927) proposed seems to fit well here. He suggested that the primary responsibility for attempting change should be put upon those who suffer the problems. Those who suffer must organize, protest, and propose change. According to him, cultural evolution is not pulled by a supreme beneficent power, but rather pushed by the experimental testing of innumerable small and gradual modifications.

Adaptive planning generally demands much more time than the expedient procedures of current practices, but that time is easily dwarfed by the time needed to mainstream procedures within the institutional layers, which is an area that, in particular, has attracted the Dutch scholars (Kemp et al. 2007; Rotmans and Loorbach 2009; Grin et al. 2011; Loorbach et al. 2017). They have found that those processes generally require 20 years or more. However, during the COVID-19 pandemic, we have seen that certain transformations can take place far more rapidly.

### Resistance to Change is High but Transformation Is Still Possible

It is indeed worrying that so many water managers are still stuck in a paradigm that prevents them from making adequate responses to the challenges that now line up in the sector. This seems to be a persistent problem, in line with the British archaeologist V. Gordon Childe’s (1936) conclusion that humans ”cling passionately to old traditions and display intense reluctance to modify customary modes or behaviour, as innovators at all times have found to their cost. The dead-weight of conservatism, largely a lazy and cowardly distaste for the strenuous and painful activity of real thinking, has undoubtedly retarded human progress….”

New conditions, marred by complexity and uncertainty (Maier et al. 2016; Madani and Shafiee-Jood 2020; Moallemi et al. 2020; Reed et al. 2022), require effective engagement, rather than avoidance by means of blueprints or other inappropriate framing choices. More than ever, it’s now a must to create the conditions for change to happen. These need to focus on providing opportunities for learning, that help actors develop adaptive expertise and individual capacity, to adequately and flexibly cope with new situations. Processes of sense-making are also essential features in governance of complex social-ecological systems. This requires a “helicopter view” rather than an ambitious penetration of minute details.

Henry Mintzberg dedicated his book, “Rebalancing Society” (2015), to those from whom we have borrowed this Earth, in the hope that they will be smarter than we have been. He asked if we hadn’t had enough of the exploiting of the world’s resources, including ourselves as “human resources”? He claimed that the world we live in is in dire needs of a radical renewal, of a kind unprecedented in human experience. According to him, the world is seriously out of balance, as too many entitlements and privileges have been given to the private sector and corporations, making them become the major rulers of it, a situation that Korten anticipated already in 1995. He concluded that we really need to engage in social movements and social initiatives, to challenge these destructive practices, asserting that we all need to mobilize our resourcefulness as human beings, to be of service to our descendants and the planet.

Nowotny et al. (2001) suggested that our reality has become more fluid, and the market has intruded into the arts, health and education sectors. Even science has been affected, as has most other sectors. As evident from systems thinking, the criteria of knowledge’s relevance are now, more than ever, related to the contexts of application. ‘Sound science’, produced away from the realities and disturbances of society has lost its legitimacy.

The future is nothing that we can masterplan into existence. It has to be shaped by means of recursive development and testing of strategies for advancement of society. Within the water sector, this translates to AWM. Yet, it seems that, so far it is mainly smaller and agile institutions, that have adopted AWM. The larger institutions have, as Allouche (2016) indicated, not fully given up their old ideas.

A turning point may have been created by the COVID-19 crisis, that broke out in late 2019. It forced the global economy into a marked slowdown and prompted people to virtually shun face-to-face-contacts with each other. The world started to partly fall apart, and governments doled out previously unseen amounts of money, just to have the show go on. The pandemic exposed the ills that forty years of neoliberal governance had brought about, and the huge spending it caused was a fundamental breach of the neoliberal dogma. The evidence of the lack of state capacity to govern under extreme conditions was very obvious, at least in the Western world. Thus, there is now a growing public demand that governments must not attempt to go back to “normal” after the pandemic, they must “build back better” or, as some express it - “never again normal”. The good news is that the COVID-19 pandemic, which craves much of our attention and resources, seems to have created a marked shift of the public sentiment, and more people are now likely to answer “Yes” to the question posed by Mintzberg. The pandemic has also demonstrated that social changes can happen much more rapidly, when challenges are serious enough. Essentially, each crisis provides invaluable opportunities to implement radical reforms by creating a common sense of importance and urgency (Madani 2020) that subsequently reduce the political cost of reforms (Madani 2019).

We are living in the midst of a deep shake-up. Thus, we cannot predict its outcome. Therefore, it is essential that we start to upgrade the systems and the intellectual tools, whereby the process of change can be managed in the best interest of humanity and the global environment. The democratization of this aspect of science is essential to make us able to muster a determined effort to achieve a system which, despite its inefficiencies, can be the most effective means for avoiding the disasters that would result from a continuation of the, far too long, stifling of criticism and the marketization of key governance functions.

## Conclusion

The world is in a predictably unpredictable state. That’s something that we urgently need to adapt to, which calls for more agile institutions. The water sector is, however, stuck in a dated paradigm, and governance systems have been hollowed out as the neoliberal ideas have spread across the globe. Although water is absolutely needed for the survival of humans and ecosystems, water issues have most of the time been pushed down the list of priority actions. In a global^i^ perspective, this has had disastrous consequences. These disasters call the water management community to fully wake up, and start addressing the challenges ahead, including the impacts of climate change. The water management community must learn to learn, both from its experience, and from external events. It needs to develop a strategic intelligence capacity and catch up with scientific advances. This is urgent as water is one of our most important resources. Without water there can be no life.

Sustainable development calls for a new development paradigm – a rebalancing of society to cater for a multitude of interests. We must no longer entrust the management and governance of water resources solely to experts. Extended peer groups are essential for a holistic approach to the problems ahead. We badly need to focus on promoting a systems-based governance structure, that serves to strengthen the capacity of management systems and people, helping them to imagine, adapt, and jointly produce a sustainable and equitable future.

From a water perspective, AWM systems, with their extension of peer communities and corresponding extension of facts, appear to be necessary for the ability to meet the new challenges related to water governance and the climate problems. They are solidly based in current scientific understanding and respect the principles of sustainable development. They are well suited to be of help in a necessary reconsideration of our framing of the problems, through an acceptance of both simple (characterized) and deep (uncharacterized) uncertainties, and a welcoming of diversity.

Yet, the rigidity of governance systems is considerable, and to be fully effective, AWM needs to be able to “trickle up” through the governance system. Thus, its success, ultimately, depends on the ability to transform the institutional overburden, so that it adapts its rules and procedures to allow the input, developed on the ground, to trickle up in a meaningful way, to make an impact, all the way up to the highest echelons. Such a paradigm shift would normally be met with suspicion or even resistance, but now, there are some hopeful signs that the tide could turn, partly thanks to the inspiring SDGs, but also due to the pandemic and the precarious state of the world.

## Data Availability

The manuscript does not have any supplementary data or materials. All the information used in the analysis is included in the manuscript and its references.
